# Sneak peek: food, waste and packaging characteristics of South Australian school children’s lunchboxes

**DOI:** 10.1017/S1368980025000126

**Published:** 2025-02-03

**Authors:** Neha Kishan Lalchandani, Clare Hume, Lynne Giles, Shona Crabb, Jo Hendrikx, Caroline Miller

**Affiliations:** 1 School of Public Health, University of Adelaide, Adelaide 5005, Australia; 2 Deakin University, Global Centre for Preventive Health and Nutrition (GLOBE), Institute for Health Transformation, Geelong, VIC 3220, Australia; 3 KESAB Environmental Solutions, 214 Grange Rd, Flinders Park, Adelaide 5025, Australia; 4 Health Policy Centre, South Australian Health and Medical Research Institute (SAHMRI), Adelaide 5000, Australia

**Keywords:** Child, Waste, Packaging, Preschools, Schools, Lunchbox, Australia

## Abstract

**Objective:**

To characterise children’s lunchbox contents for food, waste and packaging.

**Design:**

A cross-sectional study was conducted. Lunchboxes were photographed at two time points on the same day: before first morning break to capture food and packaging and post-lunch break to capture food waste. Contents were coded using an audit tool developed using REDCap.

**Setting:**

Twenty-three sites across metropolitan Adelaide, South Australia including fourteen preschools and nine primary schools in low (*n* 8), medium (*n* 7) and high (*n* 8) socioeconomic areas.

**Participants:**

Preschool (ages 3–5 years) to Grade 7 primary school (ages 6–13 years) students.

**Results:**

673 lunchboxes were analysed. Grain foods dominated (with at least half of them being discretionary varieties), with 92 % of lunchboxes having at least one item from that category, followed by fruits (78 %), snacks (62 %), dairy (32 %) and vegetables (26 %). Lunchboxes of preschool children contained more fruits (92 % *v*. 65 %; χ2(1) = 73·3, *P* < 0·01), vegetables (36 % *v*. 16 %; χ2(1) = 34·0, *P* < 0·01) and dairy items (45 % *v*. 19 %; χ2(1) = 53·6, *P* < 0·01), compared to lunchboxes of primary school children. Snack foods were more prevalent in primary school (68 %) than preschool (55 %; χ2(1) = 11·2, *P* < 0·01). Discretionary foods appeared more frequently, and single-use packaging accounted for half (53 %) of all packaging in lunchboxes, primarily from snacks and grain foods. Preschool children had less single-use packaging but more food waste. Vegetables were the most wasted food group.

**Conclusions:**

Sandwiches, fruits and various snacks are typical lunchbox foods, often accompanied by single-use packaging. Considering both health and environmental factors in lunchbox choices could benefit children and sustainability efforts in schools.

Overall dietary quality among Australian children and adolescents has been found to be nutritionally inadequate because of overconsumption of discretionary foods and underconsumption of core foods^(1,2)^. Discretionary foods and beverages such as sugar-sweetened drinks, sweet baked goods and savoury snacks that are high in sugar, fat and salt^(3)^ account for more than one-third of total energy intake among those aged 2–18 years^(1,2)^. National health survey data from 2018 indicated that, while 73 % of children met the daily recommendation for two serves of fruit, only 6 % met the recommended number of serves of both fruits and vegetables^(4)^. Moreover, it is imperative to recognise the significance of schools in shaping the dietary habits and food consumption behaviours of children, given the amount of time they spend at those educational settings.

In Australia, most school children bring a packed lunch from home^(5)^ and this school food model is also found in Norway^(6)^, Denmark^(7)^, the Netherlands^(8)^ and Canada^(9)^. Australian children consume approximately one-third of their daily energy intake at school and 44 % of this is from discretionary items^(10)^. Prior research has consistently highlighted the overrepresentation of energy-dense foods^(5)^, energy-dense and micronutrient-poor snacks (‘junk food’)^(11)^, ‘extra’ (energy-dense) foods and drinks^(12)^ or extras (food that is low nutritional value and/or high in added fat, salt or sugar)^(13)^, in Australian children’s school lunchboxes. Sanigorski et al.^(11)^ also identified that on average, a school lunchbox contained 3·1 servings of ‘junk food’ and Brennan et al.^(13)^ reported over 28 % of lunchboxes contained two or more servings of ‘extras’. These trends have stayed consistent as per studies published more recently which have characterised lunchbox contents in large Australian samples to confirm the over-representation of discretionary items in children’s lunchboxes, both in the early childhood education settings^(14)^ and primary schools^(10,15)^.

Discretionary or non-core foods are intrinsically low in nutritional quality, industrially produced, hyper-palatable products and accountable for displacing more nutritious or core food items^(3)^. There is an emergent field of food classification that focusses levels of processing (the NOVA system), and there is overlap between discretionary foods and ultra-processed foods (UPF)^(16,17)^. Consideration of the level of processing is beyond the scope of this paper, except to note that inadvertently, UPF have a range of environment-degrading effects^(18)^, and one that stands out is their single-use packaging. Seferidi et al.^(19)^ argue that while packaging allows for UPF to be mass-produced, transported over long distances and stored for long term, it is the avoidance of these foods in the first place, given they are ‘nutritionally unnecessary’, which will thereby decrease the environmental burden caused by excess food packaging.

While overconsumption of nutritionally inadequate discretionary foods in schools has been observed previously^(5,10–15)^, the literature lacks assessment of the amount and nature of packaging waste in lunchboxes that are synonymous with the consumption of those foods. There have been some USA studies which have audited food and packaging waste in the school cafeteria in the context of rising environmental concerns with the aim to divert school food waste from landfills^(20–22)^. However, lunchbox food waste assessment has been a gap identified in previous research^(12,13,23)^. Whether children prefer to eat certain types of food and thus leave others uneaten will shed light on their food choices and provide insights into how closely their consumption patterns align with previously reported dietary trends and national guidelines. Considering Lalchandani et al’s^(24)^ findings of stronger presence and implementation of food policy in preschools compared to primary schools, differences in the lunchbox contents of the two school cohorts are worth examining.

In light of both the health and environmental considerations relating to children’s school lunchbox foods, the objectives of this observational study are: first, to conduct a current assessment of the food contents of packed lunches of preschool and primary school children; and second, to assess packaging and food waste associated with these lunchboxes. Overall, this study aims to quantify and characterise the types of food brought from home to school by preschool and primary school children, how they are packaged and how much food is wasted.

## Method

### Study design and setting

This cross-sectional study involved observational audits of children’s lunchboxes in government preschools (ages 3–5 years) and primary schools (ages 6–13 years)—hereafter ‘schools’ unless comparisons made—in metropolitan Adelaide, South Australia. Demographic data collected were limited to school type (i.e. preschool or primary school), area-level socioeconomic status of schools (i.e. low, medium and high), class year level (i.e. grade), and age range of students in the class. Socio-economic status (SES) was derived from the Index of Relative Socioeconomic Advantage and Disadvantage for Australia sourced from the Australian Bureau of Statistics^(25)^.

Data collection was undertaken between March and September 2021. In this study, we audited each school only once to minimise the burden on schools, especially during the challenging period of COVID-19. This approach allowed us to maximise the number of sites sampled, ensuring representation from both preschools and primary schools across varying socioeconomic strata. The individual schools were given the authority to choose the specific dates for the audits, during which they would invite the researcher(s) based on a schedule that suited and was convenient for the school staff. None of the audits were conducted nearing the school holiday period, but the presence of COVID-19 restrictions and the prevalence of fruit fly outbreaks (predominant between early April and early June), created practical impediments which extended the data collection window across three seasons. Further detail regarding when each site was audited, along with school type, socio-economic status and fruit fly outbreak status can be found within online supplementary material, Supplemental I.

### Recruitment procedure

Schools were recruited via convenience sampling and purposive sampling to ensure a spread of SES and school type. Schools were identified for invitation to participate either through prior connections of the school with Keep South Australia Beautiful (KESAB) *environmental solutions* or cold emailing followed up with cold calling. KESAB *environmental solutions* is a non-government organisation delivering community-based environmental sustainability education programs. KESAB *environmental solutions* was an industry partner for this study.

In the first instance, school administrators were emailed the relevant information and requested to seek participants to be involved in the study from their respective schools. Thus, they forwarded the study details to the school principal or class teacher(s) whom they considered might be interested. Project information sheets and consent forms were included at this initial contact stage. Sites that confirmed participation interest were then asked to provide children with a project flyer to take home to parents or guardians. The flyer outlined the project details and explained the nature and intent of the study. An opt-out form was provided if they did not wish for their child(ren) to participate, except for one preschool that requested for parents to be provided with consent forms instead.

In preschools, the entire group present on the date of audit was included in the study (excluding children whose parents declined participation). In primary schools, two classes from each school were selected to participate. The selection of classes was undertaken by the school. Schools were informed of the audit date and staff were requested not to inform children or parents on which date the audit would take place to reduce the likelihood of them changing their usual behaviour in terms of what they pack in their child(ren)’s lunchboxes^(12,13,23)^.

### Data collection procedure

This study involved an observational method of data collection, whereby lunchbox contents were recorded using photographs by NKL, similar to the protocol by Hubbard et al.^(23)^, the difference being that food contents remained in lunchboxes instead of being spread on to a placemat by the participants and they were not asked any additional information regarding their food. The lunchbox audit took place exclusively on a single day, and children were asked to place their lunchbox wherever the school preferred to conduct the audit (either outdoors on a mat or on their desk in class). They were requested to take the lids off containers and unwrap any opaque packaging (such as aluminium foil or paper bags). Children were also asked not to dispose of any uneaten food during the day. Individual lunchboxes were photographed twice: first, at the beginning of the school day or just before snack/fruit time in the preschools or recess time in the primary schools to capture the total contents of lunchboxes (Time 1, pre-consumption); and second, at the end of the lunch break (Time 2, post-consumption). Lunchboxes were visually unique and photographs were invariably taken in order of each child’s desk location in the classroom, so the matching was done accordingly. Although the lunchbox photographs were the primary source of data for this study, additional notes were taken describing certain food and beverage items in case they were not clearly captured in the photograph.

The number of students present in class on the day of data collection, the number of lunchboxes and number of students with canteen orders were also recorded. Lunch orders and purchases made from the school canteen were not studied. The information sheet specified clearly that the research did not intend to report individual student’s or school’s data; instead, broad and anonymous food, waste and packaging data would be reported. Accordingly, no personal identifiers were collected. Any identifiers on lunchboxes (such as names stickers on lunchboxes or bags) were blurred.

### Data coding

A survey was designed using REDCap^(26,27)^ to code photo-based lunchbox data, details of which have been reported elsewhere (Author’s manuscript currently under review). In summary, lunchbox photographs were coded for presence and/or absence of the food and beverage category, followed by coding for specific items within the category. Eight food and beverage categories were used, based on the five core food groups from the Australian Guide to Healthy Eating^(28)^ with an additional three categories for common lunchbox food items mainly snacks, mixed meals such as leftovers and other beverages. Food and beverage items were categorised based on their predominant nutritional composition. The list of food items that constituted these eight broad categories was created based on the AUSNUT food nutrient database, prepared to support the 2011–13 Australian Health Survey^(29)^, and is the key methodological framework underpinning the reporting of lunchbox contents in this study. The major groupings of items within eight broad categories were also classified as predominantly core or discretionary status, based on the list of foods and the ‘discretionary’ flag attributed for the preliminary analysis of the 2011–12 National Nutrition and Physical Activity Survey data^(30)^.

Food waste was also broadly coded for each item in the lunchbox. In this study, we did not use the quarter waste method (visual estimates of none, ¼, ½, ¾, or all of a food item is wasted), although it is a common method of assessing food waste in school cafeterias^(31)^. This was because during the pilot trial (Author’s manuscript currently under review) it became evident that coding errors were frequent when there were five categories. Hence, as per Time 2 photos (after lunchbreak was over and eating time ended), food waste was categorised as ‘no waste’ (all food consumed), ‘some waste’ (partially consumed), ‘all waste’ (not consumed at all), unidentifiable (food item hidden under something or in opaque container) or missing data (lunchbox photo unavailable). Note – Children were asked to leave any uneaten items in their lunchboxes. Hence, if a packaged food item was missing in Time 2 photo, it was coded as ‘no waste’ as children were habituated to throw packaging away. The same assumption was made for whole fruits as most children discarded scraps (e.g. apple cores and banana peels) in compost bins if available at school.

The packaging for a particular food and beverage item was also coded for presence and absence, and subsequently coded for description. Note – the category of reusables does not include coding of main bento-style/compartmentalised lunchboxes or insulated/non-insulated lunch bags within which the main lunchbox or loose food items and separate/individual containers are placed, whereas small separate reusable containers were coded as reusables. Unpackaged food items placed in the main bento-style compartmentalised lunchboxes were coded as ‘Packaging absent’, while packaged food items whether inside or outside of the main lunchbox were coded as ‘Packaging present’ and the type and sub-type of packaging was also coded. Table 1 outlines the various categories of food and beverage, waste and packaging coded within REDCap.

**Table 1.** Categories for food and beverage, waste, and packaging coded using REDCap

Food and beverage categories	Vegetables (excludes legumes/beans) Fruits Grains or cereals (includes breads, cakes, biscuits) Protein and alternatives (includes meat, seafood, poultry, eggs, and legumes/beans) Dairy and alternatives (includes milk, yoghurt, cheese and their alternatives) Snacks (or extras) i.e. light foods eaten between regular meals and also includes many pre-packaged items Mixed meals i.e. items or dishes that contain multiple core food ingredients Drinks (excludes reusable water bottle from home)
Waste categories	No waste Some waste All waste Unidentifiable (data available, but food item underneath something or in opaque container) Missing data (post- snack/lunch photo unavailable)
Packaging categories (does not include food items in main lunchboxes that were unpackaged)	Reusables (containers children could bring food in again such as sandwich boxes and screw top containers, beeswax wraps). Organics (paper bags and wooden/bamboo cutlery, as well as ‘natural’ packaging of foods, such as fruit peels and skin or apple cores) Recyclables (cardboard or glass packaging, 10c drink containers) Single-use/Landfill (soft plastic and squeeze pouches which would typically go into landfill)

### Data analysis

Descriptive statistics were used to characterise the schools and children that took part in the study. The contents of lunchboxes (including the prevalence of food and beverage, waste and packaging categories as well as item descriptions) were summarised using counts and percentages. *χ* ^2^ tests for association were used to compare the presence of food and beverage, waste, and packaging categories by (1) school type and (2) school SES. Logistic regression models were fitted to examine the additive effect of school type and SES on students’ food and beverage choices, while also adjusting for the day of the week.

For inter-rater reliability measure, calculations were derived for presence or absence of individual food and beverage items, whereas for waste and packaging, scores were designated before deriving an estimate. For every item in each lunchbox at Time 2, waste was scored as no (score = 0), some (0·5) or all waste (1). Unidentifiable/missing values were excluded from the derivation of waste score during analysis. A vegetable waste score was then calculated for each lunchbox by adding together the item waste scores and then dividing by the number of vegetable items in the lunchbox. In this way, an average vegetable waste score was derived, with higher scores indicating more waste. Similar derivations were used for the fruit, grain, protein, dairy, mixed, snack and drink group items, and a total waste score across all eight major groups of lunchbox contents was then calculated by adding together the component waste scores. A packaging score for each lunchbox was calculated in a similar way. Each item in a food group was scored as having no packaging (score = 0), reusable packaging (0·25), organic packaging (0·5), recyclable packaging (0·75) or single-use packaging (1). An average packaging score for each food group was then calculated by adding together the packaging scores and dividing by the number of items in that food group in the lunchbox. A total packaging score across the eight major groups was then calculated, with possible values ranging between 0 and 8, such that higher scores indicated less desirable packaging (such as more prevalent single-use packaging).

The primary researcher (NKL) coded all lunchbox photos, and 10 % of the photos were randomly selected and coded by another researcher (JH) to evaluate reliability. The inter-rater reliability between the two coders was assessed using intraclass correlation coefficients (ICC) and 95 % confidence intervals (CIs). ICC values >0·90, between 0·75 and 0·9, between 0·5 and 0·75 and <0·5 were indicative of excellent, good, moderate and poor reliability, respectively^(32)^.

Statistical analyses were carried out using the STATA/MP version 17 (StataCorp).

## Results

### Sample

**Figure 1.**
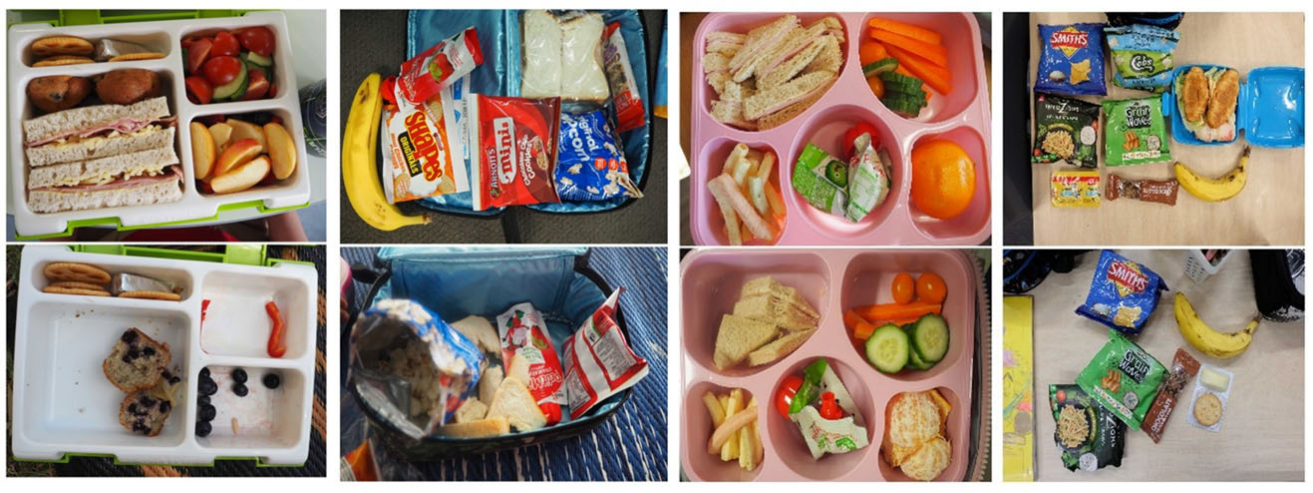
Lunchbox photos capture at two time points – Time 1 (top row) and Time 2 (bottom row).

A total of 111 sites were invited to participate (thirty-five preschools and seventy-six primary), of which twenty-three sites agreed to be involved (21 % consent rate). This included fourteen preschools and nine primary schools in a range of socioe-conomic areas (eight low SES – 4 preschools and 4 primary; 7 medium SES – 5 preschools and 2 primary and 8 high SES – 5 preschools and 3 primary). Table 2 shows the sample characteristics. Out of the total sample of 728 children, only fourteen parents declined participation (1·9 % opt out rate). A total of 681 lunchboxes were photographed, suggesting 93·5 % of children brought a packed lunch from home on the day of the study. The analysis included 673 (87·1 %) lunchbox photographs, as initial (Time 1) photos were absent for eight lunchboxes (1·2 %). Time 2 photos were absent for 11·7 % of the sample, either due to non-attendance of child (*n* 52) or because children consumed all their food before Time 2 photos were taken (*n* 28). Nevertheless, Time 1 photos for these eighty lunchboxes were still coded for food/beverage and packaging attributes, but waste was coded as missing data. Figure 1 demonstrates examples of lunchbox photos captured at both time points.

**Table 2.** Characteristics of the sample of children and lunchboxes included in the audit analysis

Characteristic	Preschool (*n* 14)	Primary school (*n* 9)
Grade	n/a	1–7
Age range (years)	3–5	6–13
Children present (*n*)	347	381
Total lunchboxes (*n*)	343	338
Lunchbox photos captured at Time 1 and 2 (*n*)	311	282
Lunchbox photo missing at Time 1 or Time 2 (*n*)	32^*^	56^*^
No lunchbox (*n*)	0	7
Lunch order (*n*)	1^**^	50^**^
Parents opted-out (*n*)	4	10

* 3 preschool and 5 primary school lunchboxes were excluded from analysis due to missing Time 2 photos.

** 1 preschool child and 26 primary school children who had a lunch order also brought a lunchbox packed from home.

### Lunchbox contents

#### Prevalence of food and beverage category

The prevalence of foods and beverages from different categories is shown in Table 3, by school type (preschool, primary school) and area-level socioeconomic status (low, medium and high). This has been reported as the percentage of total lunchboxes (*n* 673) that contained at least one item from each of the food and beverage categories to indicate presence/absence. For the whole sample (*n* 673), grains or cereals appeared in 92·4 %, fruits in 78·3 %, snacks in 61·5 %, dairy in 32·2 %, vegetables in 25·9 %, protein in 9·2 %, drinks (other than water) in 4·6 % and mixed meals in 1·2 % of all lunchboxes.

**Table 3.** Presence of food and beverage categories in lunchboxes (*n* 673), by school type and SES

	Vegetables		Fruits		Grains or cereals		Protein		Dairy		Snacks or extras		Mixed meals		Drinks	
	*n*	%	*n*	%	*n*	%	*n*	%	*n*	%	*n*	%	*n*	%	*n*	%
Overall *n* 673	**174**	**25·9**	**527**	**78·3**	**622**	**92·4**	**62**	**9·2**	**217**	**32·2**	**414**	**61·5**	**8**	**1·2**	**31**	**4·6**
Preschool *n* 340	121	35·6	312	91·8	318	93·5	45	13·2	154	45·3	188	55·3	3	0·9	11	3·2
Primary *n* 333 n(%)	53	15·9	215	64·6	304	91·3	17	5·1	63	18·9	226	67·9	5	1·5	20	6·0
χ^2^(df)	**χ** ^**2**^ **(1) = 34·0**		**χ** ^**2**^ **(1) = 73·3**		χ^2^(1) **=** 1·2		**χ** ^**2**^ **(1) = 13·3**		**χ** ^**2**^ **(1) = 53·6**		**χ** ^**2**^ **(1) = 11·2**		χ^2^(1) **=** 0·5		χ^2^(1) **=** 2·9	
*P* value^*^	***P*** **< 0·01**		***P*** **< 0·01**		*P* = 0·27		***P*** **< 0·01**		***P*** **< 0·01**		***P*** **< 0·01**		*P* = 0·46		*P* = 0·09	
Preschool																
Low SES *n* 89	22	24·7	77	86·5	84	94·4	11	12·4	49	55·1	64	71·9	1	1·1	3	3·4
Medium SES *n* 129	46	35·7	120	93·0	115	89·1	17	13·2	52	40·3	64	49·6	2	1·6	8	6·2
High SES *n* 122	53	43·4	115	94·3	119	97·5	17	13·9	53	43·4	60	49·2	0	0	0	0
χ^2^(df)	**χ** ^**2**^ **(2) = 7·9**		χ^2^(2) = 4·5		**χ** ^**2**^ **(2) = 7·4**		χ^2^(2) = 0·1		χ^2^(2) = 4·9		**χ** ^**2**^ **(2) = 13·5**		χ^2^(2) = 1·8		**χ** ^**2**^ **(2) = 7·7**	
*P* value^†^	***P*** **= 0·02**		*P* = 0·10		***P*** **= 0·02**		*P* = 0·95		*P* = 0·09		***P*** **< 0·01**		*P* = 0·41		***P*** **= 0·02**	
Primary school																
Low SES *n* 122	12	9·8	63	51·6	108	88·5	4	3·3	23	18·9	83	68·0	1	0·8	9	7·4
Medium SES *n* 67	14	20·9	37	55·2	64	95·5	6	9·0	13	19·4	42	62·7	2	3·0	8	11·9
High SES *n* 144	27	18·8	115	79·9	132	91·7	7	4·9	27	18·8	101	70·1	2	1·4	3	2·1
χ^2^(df)	χ^2^(2) = 5·5		**χ** ^**2**^ **(2) = 26·2**		χ^2^(2) = 2·7		χ^2^(2) = 2·9		χ^2^(2) = 0·01		χ^2^(2) = 1·2		χ^2^(2) = 1·4		**χ** ^**2**^ **(2) = 8·5**	
*P* value^†^	*P* = 0·07		***P*** **< 0·01**		*P* = 0·26		*P* = 0·23		*P* = 0·99		*P* = 0·56		*P* = 0·50		***P*** **= 0·01**	

% indicates percentage of lunchboxes containing at least one item from the food and beverage category. Bold values represent statistically significant results.

* Chi square test of association between presence of food group and school type.

† Chi square test of association between presence of food group and SES category in preschool/primary school.

When comparing preschools and primary schools, lunchboxes of preschool children were significantly more likely to contain fruits (91·8 % *v*. 64·6 %; χ^2^(1) = 73·3, *P* < 0·01), vegetables (35·6 % *v*. 15·9 %; χ^2^(1) = 34·0, *P* < 0·01), dairy items (45·3 % *v*. 18·9 %; χ^2^(1) = 53·6, *P* < 0·01), and protein (13·2 % *v*. 5·1 %; χ^2^(1) = 13·3, *P* < 0·01), compared to lunchboxes of primary school children. Snack foods were more prevalent in primary school children’s lunchboxes (67·9 %) than preschool children’s (55·3 %; χ^2^(1) = 11·2, *P* < 0·01).

Socioeconomic differences for food and beverage category presence also varied between preschools and primary schools. In preschools, presence of fruit was consistent across the three socioeconomic areas, but there was a significant difference in the presence of vegetables (43·4 % in high SES *v*. 35·7 % in medium SES *v*. 24·7 % in low SES; χ^2^(2) = 7·9, *P* = 0·02). Preschool children belonging to the most socio-economically disadvantaged areas had significantly more snack foods in their lunchboxes compared to their socio-economically advantaged counterparts (71·9 % in low SES *v*. 49·6 % in medium SES *v*. 49·2 % in high SES; χ^2^(2) = 13·5, *P* < 0·01). In primary schools, fruits were notably more prevalent in higher socioeconomic primary schools (79·9 % in high SES *v*. 55·2 % in medium SES *v*. 51·6 % in low SES; χ^2^(2) = 26·2, *P* < 0·01).

Table 4 displays Odds Ratio (OR) and 95 % CI from logistic regression models examining the likelihood of various food and beverage categories (outcome variables) being present in lunchboxes. Explanatory variables include school type (primary *v*. preschool), school socio-economic status (medium or high SES *v*. low SES) and the day of the week (Monday as the reference category). Vegetable consumption was significantly lower among primary school students compared to preschoolers (OR = 0·35, 95 % CI (0·23, 0·54), *P* < 0·01), with a noticeable peak in consumption on Mondays compared to Tuesdays (OR = 0·50, 95 % CI (0·25, 0·99), *P* = 0·05). Similarly, fruit consumption was also lower in primary school students compared to preschoolers (OR = 0·17, 95 % CI (0·10, 0·29), *P* < 0·01), but students from high SES schools had significantly higher odds of consuming fruits (OR = 2·57, 95 % CI (1·34, 4·94), *P* = 0·01). Day of the week did not significantly influence fruit consumption. Preschoolers were more likely to consume protein compared to primary school students (OR = 0·33, 95 % CI (0·17, 0·62), *P* < 0·01), and similarly for dairy (OR = 0·27, 95 % CI (0·18, 0·40), *P* < 0·01). Primary school students had greater odds of consuming snacks (OR = 1·59, 95 % CI (1·10, 2·30), *P* = 0·01), with a peak in consumption on Tuesday (OR = 2·18, 95 % CI (1·15, 4·13), *P* = 0·02) and Thursday (OR = 1·87, 95 % CI (1·15, 3·04), *P* = 0·01). Snacks were more common in low SES schools *v*. medium SES (OR = 0·52, 95 % CI (0·29, 0·91), *P* = 0·02). No significant associations were found between school type or SES for the consumption of grains or cereals, mixed meals and drinks.

**Table 4.** Logistic regression results for the association between school type, school socioeconomic status (SES), and day of the week with the presence of food and beverage categories in students’ lunchboxes

Food category	Comparison	OR	95 % CI	*P*-value
Vegetables	Primary *v*. Preschool	0·35	0·23, 0·54	**< 0·01**
	Medium SES *v*. Low SES	1·42	0·74, 2·71	0·29
	High SES *v*. Low SES	1·55	0·80, 2·99	0·19
	Tuesday *v*. Monday	0·50	0·25, 0·99	**0·05**
	Wednesday *v*. Monday	0·69	0·37, 1·26	0·23
	Thursday *v*. Monday	0·78	0·46, 1·30	0·34
	Friday *v*. Monday	0·53	0·22, 1·31	0·17
Fruits	Primary *v*. Preschool	0·17	0·10, 0·29	**< 0·01**
	Medium SES *v*. Low SES	1·23	0·65, 2·32	0·53
	High SES *v*. Low SES	2·57	1·34, 4·94	**0·01**
	Tuesday *v*. Monday	0·70	0·22, 2·19	0·54
	Wednesday *v*. Monday	0·63	0·28, 1·39	0·25
	Thursday *v*. Monday	0·53	0·24, 1·14	0·11
	Friday *v*. Monday	0·45	0·17, 1·19	0·11
Grains	Primary *v*. Preschool	0·83	0·42, 1·62	0·58
	Medium SES *v*. Low SES	0·88	0·35, 2·23	0·79
	High SES *v*. Low SES	1·32	0·50, 3·49	0·58
	Tuesday *v*. Monday	0·91	0·24, 3·50	0·89
	Wednesday *v*. Monday	0·58	0·20, 1·67	0·31
	Thursday *v*. Monday	0·67	0·25, 1·82	0·43
	Friday *v*. Monday	0·57	0·15, 2·24	0·42
Protein	Primary *v*. Preschool	0·33	0·17, 0·62	**0·00**
	Medium SES *v*. Low SES	1·05	0·39, 2·78	0·93
	High SES *v*. Low SES	0·93	0·34, 2·58	0·89
	Tuesday *v*. Monday	0·40	0·13, 1·23	0·11
	Wednesday *v*. Monday	0·77	0·30, 1·98	0·59
	Thursday *v*. Monday	1·11	0·53, 2·32	0·79
	Friday *v*. Monday	0·75	0·20, 2·79	0·67
Dairy	Primary *v*. Preschool	0·27	0·18, 0·40	**< 0·01**
	Medium SES *v*. Low SES	0·89	0·49, 1·59	0·68
	High SES *v*. Low SES	1·08	0·60, 1·95	0·80
	Tuesday *v*. Monday	1·76	0·93, 3·33	0·08
	Wednesday *v*. Monday	1·21	0·66, 2·22	0·54
	Thursday *v*. Monday	1·43	0·85, 2·41	0·18
	Friday *v*. Monday	1·92	0·86, 4·25	0·11
Snacks	Primary *v*. Preschool	1·59	1·10, 2·30	**0·01**
	Medium SES *v*. Low SES	0·52	0·30, 0·91	**0·02**
	High SES *v*. Low SES	0·72	0·41, 1·27	0·26
	Tuesday *v*. Monday	2·18	1·15, 4·13	**0·02**
	Wednesday *v*. Monday	1·36	0·79, 2·34	0·26
	Thursday *v*. Monday	1·87	1·15, 3·04	**0·02**
	Friday *v*. Monday	1·22	0·57, 2·61	0·60
Mixed items	Primary *v*. Preschool	2·28	0·36, 14·30	0·38
	Medium SES *v*. Low SES	1·41	0·24, 8·39	0·71
	High SES *v*. Low SES	0·45	0·05, 3·87	0·47
	Tuesday *v*. Monday	1·81	0·09, 36·48	0·70
	Wednesday *v*. Monday	1·97	0·16, 25·05	0·60
	Thursday *v*. Monday	0·83	0·06, 11·85	0·89
	Friday *v*. Monday	N/A	N/A	N/A
Drinks	Primary *v*. Preschool	1·87	0·79, 4·43	0·15
	Medium SES *v*. Low SES	3·08	0·83, 11·51	0·09
	High SES *v*. Low SES	0·31	0·06, 1·75	0·19
	Tuesday *v*. Monday	N/A	N/A	N/A
	Wednesday *v*. Monday	1·32	0·34, 5·12	0·69
	Thursday *v*. Monday	0·60	0·15, 2·38	0·46
	Friday *v*. Monday	2·00	0·31, 12·90	0·47

Odds Ratio (OR) | Confidence Interval (CI) | *P*-values for statistically significant results are highlighted in bold | ‘N/A’ indicates results that could not be calculated due to insufficient sample size.

#### Diversity of food and beverage items

**Figure 2.**
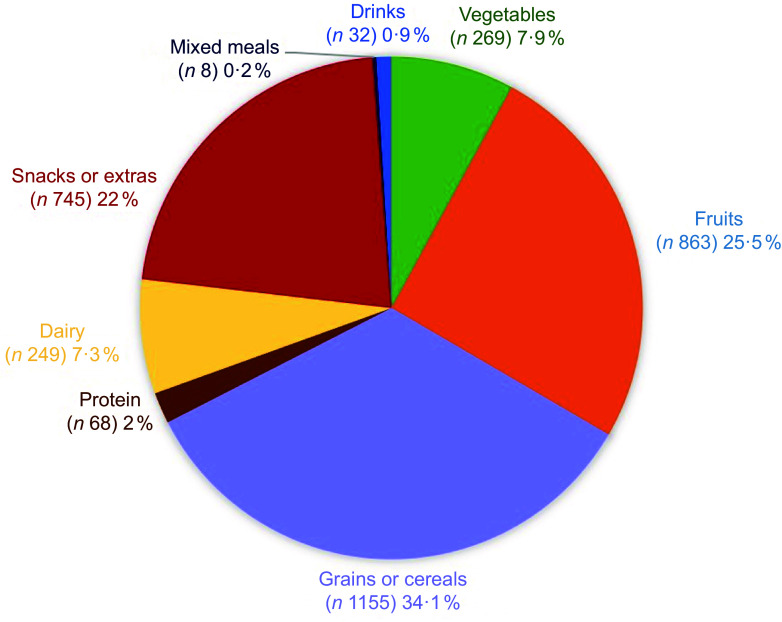
Proportion of different food groups observed based on total number of food items (*n* 3389) in 673 lunchboxes.

There was a total of 3389 individual food/beverage items in the lunchboxes, and the proportion of items in each category is presented in Figure 2. Of this total, grain food items were the most common (34·1 %) followed by fruits (25·5 %) and snacks (22 %). Table 5 provides details of the variety of items within each group that were frequently observed in the lunchboxes, along with the broad classification according to core or discretionary status for each grouping of items. For vegetables, cucumbers, carrots and cherry tomatoes were the most common. For fruits, the top three were apples, bananas and mandarins; but there was three times more variety in the types of fruits when compared to vegetables. Out of the 269 vegetables, 249 (92·6 %) were fresh and the remaining 7·4 % were either cooked, fried or oven-baked or preserved (i.e. dried, fermented, picked). Out of the 863 fruits, 783 (90·8 %) were fresh, while the other 8·2 % were either canned or preserved, tub or diced, pureed or dried. For grains or cereals, core items such as sandwiches/rolls/wraps were frequently included, accounting for 37·7 % of the total. Savoury biscuits/crackers and sweet biscuits/baked goods represented a significant portion, comprising 49·9 % of all grains. In the snacks category, discretionary items, such as potato chips (crisps), muesli bars and grain/legume-based snacks, made up two-thirds of the category. Protein options in lunchboxes were limited to discretionary choices, with processed and crumbed meat products accounting for 85·3 % of the protein sources. Dairy were limited in variety, with yoghurt and cheese being the predominant core, making up 81·1 % of the total. There were very few drink varieties or mixed meals. On average, children had one vegetable, one to two fruits, one to three grains or cereals item, one protein, one dairy and one to two snacks in their lunchboxes. Almost every child had a water bottle, so they were neither assessed nor counted as drinks.

**Table 5.** Description and frequency of food and beverage items (*n* 3389) in sample of 673 lunchboxes

List of food and beverage items within each category	n pieces	% of category	% of total items *n*	Core/Discretionary status
Vegetables (Total)	269	100·0	7·9	
Cucumbers	100	37·2	3·0	Core
Carrots	68	25·3	2·0	Core
Tomato (includes cherry variant)	44	16·4	1·3	Core
Others (e.g. capsicums, snow peas, celery, corn, dried seaweed, etc)	57	21·2	1·7	Core
Fruits (Total)	863	100·0	25·5	
Apples	186	21·6	5·5	Core
Bananas	154	17·8	4·5	Core
Citrus fruit (i.e. mandarin)	141	16·3	4·2	Core
Berry fruit (i.e. strawberries)	108	12·5	3·2	Core
Others (e.g. fresh grapes, watermelon, dried grapes, oranges, blueberries, etc)	274	31·7	8·1	Core
Grains or cereals (Total)	1155	100·0	34·1	
Regular breads, sandwiches, rolls, wraps, flat breads	435	37·7	12·8	Core
Savoury biscuits and crackers (flour or wholegrain based)	244	21·1	7·2	Core/Discretionary^*^
Sweet biscuits, cookies, crackers, and wafers	176	15·2	5·2	Discretionary
Sweet baked products (e.g. cakes, muffins, slices, breads, buns, scrolls, doughnuts, pancakes)	157	13·6	4·6	Discretionary
Others (e.g. savoury topped breads, pastry products, fast food items, pasta and rice dishes, etc)	143	12·4	4·2	Core/Discretionary^*^
Protein (Total)	68	100·0	2·0	
Processed meats (e.g. bacon, ham, salami, meatballs, fritz, sausages)	47	69·1	1·4	Discretionary
Crumbed meat product (e.g. nuggets)	11	16·2	0·3	Discretionary
Others (e.g. eggs, canned tuna, soybean products)	10	14·7	0·3	Core
Dairy (Total)	249	100·0	7·3	
Flavoured yoghurt	121	48·6	3·6	Core
Cheese (i.e. hard and soft varieties)	81	32·5	2·4	Core
Others (e.g. dairy desserts, Yakult, plain yogurt, plain and flavoured milk, etc)	47	18·9	1·4	Core/Discretionary^*^
Snacks or extras (Total)	745	100·0	22·0	
Potato or other vegetable chips/crisps/puffy snacks	191	25·6	5·6	Discretionary
Grain, cereal, fruit, nuts, or seeds bars	183	24·6	5·4	Discretionary
Grain or legume-based snacks/crisps/chips (includes popcorn)	127	17·0	3·7	Discretionary
Others (e.g. fruit leathers and straps, cheese and cracker snack packs, confectionery items, etc)	244	32·8	7·2	Discretionary
Mixed meals (Total)	8	100·0	0·2	
Rice with vegetables/meat/egg, filled taco, soup	8	100·0	0·2	Core/Discretionary^*^
Drinks (Total)	32	100·0	0·9	
Fruit or vegetable juices (reconstituted, made from concentrates, or with added sugar)	21	65·6	0·6	Discretionary
Others (e.g. breakfast cereal beverages, plain bottled water, soft drink (soda))	11	34·4	0·3	Core/Discretionary^*^

* Dual core/discretionary flagging is owing to food groupings or lack of nutritional information in the context of this study e.g. Savoury biscuits, wheat based, plain, energy > 1800 kJ per 100 g would be discretionary/Savoury pasta/noodle and sauce dishes, saturated fat > 5 g/100 g would be discretionary^(30)^.

### Food waste

Vegetables were the most wasted category (50·5 %) followed by fruits (36·8 %) and grains or cereals (34·7 %). Preschoolers, compared to primary schoolers, were shown to waste more vegetables (56·8 % *v*. 35·1 %; χ2(2) = 13·2 *P* < 0·01), fruits (38·2 % *v*. 33·8 %; χ2(2) = 10·5 *P* = 0·01), grains or cereals (46·7 % *v*. 21·4; χ2(2) = 81·8 *P* < 0·01) and snacks (39·1 % *v*. 15·2 %; χ2(2) = 66·5 *P* < 0·01). Overall, snacks, dairy and protein items were the food types most likely to be completely consumed. When examined by SES, the following differences were observed: Low SES preschoolers were more likely than high SES preschoolers to leave fruit and grain food waste. Further results are presented in Table 6.

**Table 6.** Food waste measure of food and beverage items (*n* 3389) by school type and SES

							Preschool						Primary school					
	Total items		Preschool items		Primary school items		Low		Med		High		Low		Med		High	
	*n*	%	*n*	%	*n*	%	*n*	%	*n*	%	*n*	%	*n*	%	*n*	%	*n*	%
Vegetables	269	100	192	71·4	77	28·6	28	14·6	69	35·9	95	49·5	17	22·1	18	23·4	42	54·5
No waste	110	40·9	65	33·9	45	58·4	9	32·1	16	23·2	40	42·1	9	52·9	8	44·4	28	66·7
Some waste	45	16·7	37	19·3	8	10·4	4	14·3	15	21·7	18	18·9	1	5·9	3	16·7	4	9·5
All waste	91	33·8	72	37·5	19	24·7	12	42·9	30	43·5	30	31·6	5	29·4	4	22·2	10	23·8
Unidentifiable/missing data	23	8·6	18	9·4	5	6·5	3	10·7	8	11·6	7	7·4	2	11·8	3	16·7	0	0
χ2(df) *P* value^*^^,^^†^^,^^‡^			**χ2(2) = 13·2** ***P*** **< 0·01**				χ2(4) = 6·5 *P* = 0·16						χ2(4) = 2·1 *P* = 0·71					
Fruits	863	100	573	66·4	290	33·6	119	20·8	216	37·7	238	41·5	79	27·2	47	16·2	164	56·6
No waste	435	50·4	268	46·8	167	57·6	45	37·8	102	47·2	121	50·8	48	60·8	21	44·7	98	59·8
Some waste	137	15·9	105	18·3	32	11·0	25	21·0	32	14·8	48	20·2	2	2·5	4	8·5	26	15·9
All waste	180	20·9	114	19·9	66	22·8	35	29·4	36	16·7	43	18·1	21	26·6	14	29·8	31	18·9
Unidentifiable/missing data	111	12·9	86	15·0	25	8·6	14	11·8	46	21·3	26	10·9	8	10·1	8	17·0	9	5·5
χ2(df) *P* value^*^^,^^†^^,^^‡^			**χ2(2) = 10·5** ***P*** **= 0·01**				**χ2(4) = 10·4** ***P*** **= 0·04**						**χ2(4) = 12·8** ***P*** **= 0·01**					
Grains or cereals	1155	100	608	52·6	547	47·4	161	26·5	201	33·1	246	40·5	178	32·5	108	19·7	261	47·7
No waste	666	57·7	286	47·0	380	69·5	60	37·3	98	48·8	128	52·0	130	73·0	64	59·3	186	71·3
Some waste	221	19·1	166	27·3	55	10·1	51	31·7	45	22·4	70	28·5	14	7·9	17	15·7	24	9·2
All waste	180	15·6	118	19·4	62	11·3	41	25·5	48	23·9	29	11·8	21	11·8	10	9·3	31	11·9
Unidentifiable/missing data	88	7·6	38	6·3	50	9·1	9	5·6	10	5·0	19	7·7	13	7·3	17	15·7	20	7·7
χ2(df) *P* value^*^^,^^†^^,^^‡^			**χ2(2) = 81·8** ***P*** **< 0·01**				**χ2(4) = 20·1** ***P*** **< 0·01**						χ2(4) = 6·8 *P* = 0·15					
Protein	68	100	49	72·1	19	27·9	11	22·4	19	38·8	19	38·8	4	21·1	8	42·1	7	36·8
No waste	43	63·2	33	67·3	10	52·6	7	63·6	11	57·9	15	78·9	2	50·0	3	37·5	5	71·4
Some waste	12	17·6	10	20·4	2	10·5	3	27·3	7	36·8	0	0	0	0	0	0	2	28·6
All waste	5	7·4	4	8·2	1	5·3	1	9·1	1	5·3	2	10·5	1	25·0	0	0	0	0
Unidentifiable/missing data	8	11·8	2	4·1	6	31·6	0	0	0	0	2	10·5	1	25·0	5	62·5	0	0
χ2(df) *P* value^*^^,^^†^^,^^‡^			χ2(2) = 0·2 *P* = 0·88				χ2(4) = 7·7 *P* = 0·10						χ2(4) = 5·3 *P* = 0·26					
Dairy	249	100	179	71·9	70	28·1	59	35·2	60	31·5	60	33·3	26	37·1	13	18·6	31	44·3
No waste	156	62·7	104	58·1	52	74·3	35	59·3	30	50·0	39	65·0	18	69·2	11	84·6	23	74·2
Some waste	18	7·2	13	7·3	5	7·1	4	6·8	6	10·0	3	5·0	2	7·7	1	7·7	2	6·5
All waste	55	22·1	45	25·1	10	14·3	18	30·5	15	25·0	12	20·0	3	11·5	1	7·7	6	19·4
Unidentifiable/missing data	20	8·0	17	9·5	3	4·3	2	3·4	9	15·0	6	10·0	3	11·5	0	0	0	0
χ2(df) *P* value^*^^,^^†^^,^^‡^			χ2(2) = 4·5 *P* = 0·10				χ2(4) = 3·2 *P* = 0·53						χ2(4) = 1·1 *P* = 0·89					
Snacks or extras	745	100	312	41·9	433	58·1	129	42·8	102	31·1	81	26·1	183	42·3	73	16·9	177	40·9
No waste	490	65·8	161	51·6	329	76·0	68	52·7	44	43·1	49	60·5	145	79·2	44	60·3	140	79·1
Some waste	55	7·4	45	14·4	10	2·3	16	12·4	18	17·6	11	13·6	2	1·1	1	1·4	7	4·0
All waste	133	17·9	77	24·7	56	12·9	37	28·7	26	25·5	14	17·3	17	9·3	20	27·4	19	10·7
Unidentifiable/missing data	67	9·0	29	9·3	38	8·8	8	6·2	14	13·7	7	8·6	19	10·4	8	11·0	11	6·2
χ2(df) *P* value^*^^,^^†^^,^^‡^			**χ2(2) = 66·5** ***P*** **< 0·01**				**χ2(4) = 6·2** ***P*** **= 0·19**						**χ2(4) = 20·8** ***P*** **< 0·01**					

Bold values represent statistically significant results.

* Chi squares do not include unidentifiable or missing data.

† Chi square test of association between approximate food waste amount and school type.

‡ Chi square test of association between presence of food group and SES category in preschool/primary school.

### Packaging

Overall, 38·2 % of food items were unpackaged and found in the main compartmentalised lunchboxes. Bento-style lunchboxes were more common among preschool children (54·7 %) *v*. primary children (24 %; χ^2^(1) = 66·3, *P* < 0·01), whereas lunch bags were common across both cohorts (77 % for preschool and 69 % for primary school), with some children bringing both. Table 7 provides an overview of the types of packaging (present or absent) in lunchboxes. Table 7 also presents the type of packaging that was used with different food types and items, to show the relationship between food type and packaging. Drinks, mixed meals, dairy and snacks were highly likely to be packaged, whereas fruits and vegetables were least likely to be packaged. Snacks made up the largest proportion (42·5 %) of the single-use packaging which would end up in landfill. Single-use packaging was also predominant for dairy foods and was frequently used for grains or cereals. Grains or cereals were packed in reusable containers almost as frequently as single-use packaging. Fruits and vegetables were predominantly packed in reusable containers, while all other food types, including drinks, snacks and dairy, were packed in reusable containers at least in some instances. Fruits yielded the most (non-edible) organic waste. Recyclables were the least common packaging type found in lunchboxes, with the notable exception of drinks. When comparing packaging waste trends between preschools and primary schools, the latter had a higher proportion of single-use packaging within the grains and snacks category. Preschools had more reusable containers overall.

**Table 7.** Presence of packaging category in relation to respective food and beverage categories for preschools and primary schools

						Packaging category								
	*n* items within F&B category	*n*	% items within F&B category	*n*	% items within F&B category	*n*	% items within packaging category	*n*	% items within packaging category	*n*	% items within packaging category		*n*	% items within packaging category
Food and beverage (F&B) category		Packaging absent^*^		Packaging present		Reusable^**^		Organics		Recyclables			Single use/Landfill	
Vegetables	269	183	68·0	86	32·0	67	8·7	4	0·8	1		1·3	19	1·4
Preschool	192	139	72·4	53	27·6	42		3		1			11	
Primary school	77	44	57·1	33	42·9	25		1		0			8	
Fruits	863	576	66·7	287	33·3	236	30·8	440	92·8	20		26·3	47	3·5
Preschool	573	365	63·7	208	36·3	184		239		11			21	
Primary school	290	211	72·8	79	27·2	52		201		9			26	
Grains or cereals	1155	361	31·3	794	68·7	341	44·5	27	5·7	2		2·6	486	35·7
Preschool	608	260	42·8	348	57·2	188		8		0			177	
Primary school	547	101	18·5	446	81·5	153		19		2			309	
Protein	68	37	54·4	31	45·6	17	2·2	2	0·4	0		0	15	1·1
Preschool	49	29	59·2	20	40·8	11		2		0			7	
Primary school	19	8	42·1	11	57·9	6		0		0			8	
Dairy	249	30	12·0	219	88·0	29	3·8	1	0·2	21		27·6	195	14·3
Preschool	179	25	14·0	154	86·0	24		1		10			133	
Primary school	70	5	7·1	65	92·9	5		0		11			62	
Snacks or extras	745	106	14·2	639	85·8	62	8·1	0	0	2		2·6	578	42·5
Preschool	312	86	27·6	226	72·4	34		0		0			192	
Primary school	433	20	4·6	413	95·4	28		0		2			386	
Mixed meals	8	0	0	8	100	12	1·6	0	0	0		0	0	0
Preschool	3	0	0	3	100	4		0		0			0	
Primary school	5	0	0	5	100	8		0		0			0	
Drinks	32	0	0	32	100	2	0·3	0	0	30		39·5	21	1·5
Preschool	11	0	0	11	100	0		0		11			8	
Primary school	21	0	0	21	100	2		0		19			13	
Total	3389	1293	38·2	2096	61·8	766	100	474	100	76		100	1361	100
Preschool	1927	904	46·9	1023	53·1	487		253		33			549	
Primary school	1462	389	26·6	1073	73·4	279		221		43			812	
		**χ2(1) = 145·2** ***P*** **< 0·01**												

Bold values represent statistically significant results.

* Packaging was absent because the food item was found in the main bento-style of compartmentalised lunchbox.

** Reusable containers may be counted more than once when it contained more than one food type and item in the same container e.g. in the event that one reusable container held carrot sticks, cherry tomatoes and grapes.

Table 8 lists the various packaging items within each category. There were 2569 individual items of packaging. Over half of the packaging items observed in lunchboxes were single-use/landfill packaging (53 %; *n* 1361 pieces of packaging waste), 25·6 % (*n* 658) were reusables and 18·5 % were organics (*n* 474). The most common reusable packaging was separate containers (85·3 %). Organics or compostable packaging made up 18·5 % of overall observed packaging, with food scraps (i.e. fruit peels, rinds and cores) constituting the highest proportion (92·6 %) of the organics category. Single-use packaging made up 60·2 % of packaging items in primary school children’s lunchboxes compared to 44·9 % of packaging in preschoolers lunchboxes (χ^2^(2) = 60·45, *P* < 0·01). Overall, the single-use packaging category was dominated by soft plastic or silver lined wrappers (50·7 % of the category and contributing 26·9 % of all packaging), which contained items such as chips/crisps and bars (snacks). Re-sealable (zip-lock) plastic bags and cling wrap made up 22 % of all single use packaging, and was frequently used for items such as sandwiches and wraps (grains or cereals), present in both preschools and primary schools. Squeeze pouches were a common source of single-use packaging in the preschool cohort (16·9 %) and a common type of packaging for flavoured yoghurts (dairy foods).

**Table 8.** Description and frequency measure of packaging items (*n* 2569) in sample of 673 lunchboxes

	Preschool		Primary school		Total		Overall
Packaging type	*n*	%	*n*	%	*n*	%	% of *n*
Reusables	387	100	271	100	658	100	25·6
Separate container^*^	333	86·0	228	84·1	561	85·3	21·8
Reusable cutlery	33	8·5	24	8·9	57	8·7	2·2
Stainless steel food flask	9	2·3	12	4·4	21	3·2	0·8
Others (e.g. silicone bag/cup, cloth/cotton bag, beeswax wrap)	12	3·1	7	2·6	19	2·9	0·7
Organics	253	100	221	100	474	100	18·5
Food scraps	236	93·3	200	90·5	439	92·6	17·1
Paper (wrapper/bag)	3	1·2	13	5·9	16	3·4	0·6
Paper towel or tissue	10	4·0	6	2·7	16	3·4	0·6
Others (e.g. compostable cutlery, certified compostable packaging)	1	0·4	2	0·9	3	0·6	0·1
Recyclables	33	100	43	100	76	100	3·0
Cardboard or carton	21	63·6	21	48·8	42	55·3	1·6
10 cent drink container	10	30·3	16	37·2	26	34·2	1·0
Others (e.g. hard plastic container, aluminium/steel tin or can, glass jar/bottle)	2	6·1	6	14·0	8	10·5	0·3
Single-use or landfill	549	100	812	100	1361	100	53·0
Soft plastic or silver lined wrapper	232	42·3	458	56·4	690	50·7	26·9
Plastic resealable bags	82	14·9	110	13·5	192	14·1	7·5
Mixed (≥ 2 packaging elements)	61	11·1	62	7·6	123	9·0	4·8
Squeeze pouches	93	16·9	25	3·1	118	8·7	4·6
Cling wrap	29	5·3	79	9·7	108	7·9	4·2
Muffin or cupcake case/Parchment paper	18	3·3	24	3·0	42	3·1	1·6
Foil (aluminium, paper lined)	17	3·1	21	2·6	38	2·8	1·5
Others (e.g. small plastic or condiment packaging, plastic straw, cutlery, small tins or cans, etc)	17	3·1	33	4·1	50	3·7	1·9
Total	1222		1347		2569		100 %

* This table includes the adjusted count for separate containers.

### Inter-coder reliability measure

Across the sixty-eight lunchboxes that were dual coded, 153 ICC estimates were derived for presence/absence of food and beverage items. Of the 153 values, 124 were >0·9 (excellent reliability), 4 were between 0·75 and 0·9 (good reliability), 10 were between 0·5 and 0·75 (moderate reliability), 2 were less than 0·5 (poor reliability) and 13 were not calculable due to insufficient observations (*n* 0–2) for the specific item. Vegetables and fruits constituted over half (55 %) of the excellent coding estimate, followed by snacks or extras (20 %) and grains or cereals (12·5 %). The latter two were also the predominant constituents of the moderate ICC estimate (50 % and 40 %). The ICC estimate was 0·979 (95 % CI 0·967, 0·987) for total waste and 0·976 (95 % CI 0·960, 0·985) for total packaging.

## Discussion

The current study expands the literature by incorporating an environmental dimension into standard lunchbox assessments, specifically examining the under-studied aspect of food waste and packaging. This lunchbox contents data also present an update to the most recent previous studies which were published near a decade ago^(11–13,23)^, and this South Australian data also complement more recent published research from New South Wales^(14,15)^ and nationally^(10)^. Dietary patterns of school children have often not been in alignment with dietary guidelines, and the results of this study confirm this trend. Findings from this lunchbox assessment are consistent with previous studies which showed low consumption of vegetables and high consumption of discretionary items by children in Australia and New Zealand^(5,11–13,23,33)^ and also supports results from consecutive Australian Health Surveys^(4)^. Consistent with the bin content analysis in New Zealand by Dresler-Hawke et al.^(33)^, where fruit and vegetables were mostly thrown away, waste results reported in this study affirm that children are often not consuming vegetables, even when they are sent from home and present in lunchboxes (which had occurred 25·9 % of the time). Promisingly, a high proportion of children’s lunchboxes contained fruit (78·3 %), but greater emphasis needs to be placed on vegetable consumption as well, in line with dietary guidelines. The rates of wastage of fruit and vegetables, if unconsumed by children, are likely to be a barrier to provision for many parents.

What has also remained consistent is the composition of a typical school lunch which includes a mix of core items such as sandwich and fruit and discretionary items predominantly in the form of savoury snacks and sweet treats, while mixed meals (such as leftovers) remain uncommon^(23,33)^. There is a notable and encouraging absence of sugar-sweetened beverages in our sample of preschools and primary schools in comparison to previous studies^(5,11,12,23)^. This is likely due to school-level policies actively discouraging such beverages and/or prohibiting their sale in canteens, which has a flow-on effect on social norms in the schools. Savoury snacks like potato chips (crisps) and muesli/fruit bars were common in lunchboxes. The associated environmental implications of these pre-packaged, often discretionary, foods are particularly noteworthy. These food choice patterns coincide with existing literature which notes children’s consumption trends towards pre-packaged foods. As observed by Sanigorski et al.^(11)^, children are not bringing just one but multiple snacks of these types, which has both nutritional implications and environmental implications from packaging. It is noteworthy that snack food items were among the least wasted, indicating they were being consumed by children, which potentially reinforces parents wanting to pack food that their children like, will eat, and will not result in food wastage.

As part of sustainability efforts in preschools and primary schools, Australian children are encouraged to bring ‘nude’ foods on specific days, and more commonly. This means bringing foods with either no packaging or reusable packaging only. There was higher presence of ‘nude’ or unpackaged foods in reusable containers or bento-style compartmentalised lunchboxes in preschools in comparison to primary schools. The difference is worth highlighting as it brings to the forefront the various factors influencing lunchbox packing practices within the two school types, likely due to a more robust presence of food policies in preschools as opposed to primary schools^(24)^. The transition from preschool to primary school seems to impact what children bring in their lunchboxes in terms of nutritional quality and whether foods are pre-packaged or not. This is likely to be due, at least in part to more explicit policy in place in preschools, as well as social norms in these settings and children’s preferences for certain foods. For instance, a noteworthy difference between preschool and primary school settings observed in this study was the variation in eating times and the presence or absence of teacher supervision. Preschool eating time is longer and less structured than primary schools where eating time is often reduced to 10 min and children are unsupervised by teachers during the break. There are other factors such as older children being more involved in food choices and some even packing their own lunchboxes. Despite varying circumstances, there is potential for school-based reforms such as the continuation of policies from preschools into primary schools to encourage the continued consumption of nutritious and unpackaged foods into primary year levels, keeping in mind the growing autonomy of children’s choices as they progress with age.

There were some differences in lunchbox food contents observed by socio-economic status, although these differences were more pronounced in preschoolers compared to primary school children. Vegetables and fruits were more prevalent in high SES schools. Specifically, preschool children in high SES areas had higher vegetable consumption compared to their lower SES counterparts, and primary school children in high SES areas had higher fruit consumption than those in lower SES areas. Snacks were more prevalent in preschoolers’ lunchboxes in lower SES areas, whereas no significant differences in snack consumption by SES were found for primary school children, where snacks were commonly present (61·5 %) across all SES groups. SES is associated with the prevalence of overweight and obesity in children according to Australian Institute of Health and Welfare^(34)^. Evidence suggests that low SES also has associations with the overall dietary quality among school children^(11)^, where consumption of fruits and vegetables is often compromised, hence calling for targeted health interventions there^(35,36)^. However, the dominance of pre-packaged snack foods in primary school lunchboxes, and across low and high SES areas overall, suggests that interventions should target students and schools in all areas by combining both health and environmental agendas together.

This study was able to draw tangible parallels between the types of food packed in school lunchboxes, consumed *v*. unconsumed foods that contributed to lunchbox food waste and the prevalence of various packaging types of foods and beverages in lunchboxes. There is increased recognition of the importance of addressing nutrition early in life and of healthy eating interventions directed to preschools, childcare centres and primary schools^(37–40)^. What seems to be missing is the attachment of the environmental consideration to healthy eating interventions, so the importance and connection of both agendas are realised for health promotion. One way to create positive dietary behaviour change could be to encourage an increase in the consumption of unpackaged foods and a decrease in the consumption of pre-packaged foods, which may ultimately have positive implications on health and the environment. To increase packing and consumption of unpackaged foods, targeted interventions to provide support or encouragement may be useful for lunchbox packers, either parents or children, to pack waste-free lunches, replace disposable packaging options with reusable ones, while driving consumption of more nutritious foods. Lalchandani et al.^(41)^ reviewed ten studies that considered food and packaging waste in the context of lunchboxes; the scoping review highlighted the possibility of mobilising the health and sustainability nexus by running interventions that are accessible and feasible for families to implement in their everyday life, encourage participator behaviours by children when it comes to lunchbox food choices and packing, and considering wider social influences when it comes to public health behaviours. However, whether environmental conservation in the context of lunchbox packing is a priority and the extent to which interventions or strategies are sought by parents and children need further investigation. Future research can explore what the perceived barriers are to packing lunchboxes that are in line with dietary guidelines and consist of minimal or no packaging.

### Study limitations

The current study has several limitations. This study only audited the lunchboxes of public preschool and primary school children in one state of Australia; although a majority of schools in the state are government schools, the lunchbox contents of private, faith based and independent schools’ children were not assessed. Hence, the sample of this study may not be representative of the entire Australian population. It is also worth acknowledging that, due to the self-selected nature of recruitment, schools agreeing to participate were more likely to have a higher level of environmental awareness than the general school populations. However, prior research has shown that very few schools in South Australia have policies in place regarding food and environmental issues^(24)^. Hence, considering these factors together, any bias due to convenience sampling is low.

Instead of micro-analysis of lunchbox contents where food items are weighed and recorded in detail for macro- and micro-nutrient composition, as per previously implemented protocols^(11,12,42,43)^, this study did not include any detailed accounting for food nutrient profiling and unnoticeable contents. In certain sub-categories of food, the distinction between core and discretionary items is not clear due to grouping of items or the absence of nutrient information. Sandwiches and wraps were not unwrapped or disassembled to analyse fillings, so it is anticipated that the protein group and to some extent dairy (cheese), which tend to be common sandwich fillings, is underrepresented. This approach is owing to the utilisation of an opt-out ethics process, and one of the components of that agreement was that food items would not be touched by the research team. As a result, the number of participants who were recruited outweighs the lack of micro-nutrient or sandwich fillings details.

While this study was able to measure food consumption at school, children could have consumed any uneaten food left in the lunchbox during the latter part of the school day, on their way home or at home as an afternoon snack. Hence, this food may not have been wasted as suggested by this study. There were also some limitations in collecting waste data at school, mainly because a lot of food items such as sandwiches, fruits and snacks are highly portable (allowing children to consume them on their way to play); thus, children may not have adhered to the request of leaving any uneaten foods in their lunchboxes, or they may have disposed of fruit peels and cores in the organics bins on site, meaning there was no way to determine the extent of the waste. Moreover, this study was unable to determine the fate of waste and packaging. There are multiple streams for various packaging to be recycled, but this study did not capture how the waste could have potentially been recycled and diverted from landfill (for instance, soft plastic recycling or the South Australian 10-cent container deposit scheme where drink cans and containers can be recycled in exchange for money).

It is also notable there was a fruit fly outbreak in Adelaide during 2020–2021, which interrupted data collection, particularly between early April and June when it was at its peak, compounded also by COVID-19 restrictions. At the start of the school term in the last week of January 2021, there were restrictions on which fruits and vegetables could be packed in children’s lunchboxes as fruit movement bans were announced across Adelaide. These restrictions led to some confusion but eventually, most schools navigated this impediment and lifted fruit bans while encouraging the disposal of fruit scraps on-site to restrict movement of fruit between geographical areas. The fruit fly outbreak and restrictions may or may not have caused differences in lunchbox contents during the data collection phase. Finally, data collection timeframe spanned three seasons (March–September 2021); this extended duration could have led to variations in the types of food typically included in lunchboxes (especially fruits and vegetables) due to seasonal changes.

Regardless of limitations, the reliability of the tool developed for this study was tested and indicative of mostly excellent agreement, suggesting that individual coders made consistent observations with respect to coding the lunchbox photos. While previous studies have analysed lunchbox contents, this study aimed to provide an update of Australian lunchbox contents, and it does so through a relatively large sample size (*n* 673). Additionally, it makes a new contribution by reporting the amount and nature of packaging waste in lunchboxes, while also attempting to make parallels with the nature of foods observed in lunchboxes, with the high-level core/discretionary status attributed. Lastly, this study was able to present an update on lunchbox contents data to guide future research and interventions, useful in the context of sustainability.

### Conclusions

Overall, preschoolers’ lunchboxes were nutritionally superior; however, food waste measures were high in this cohort in comparison to primary school children. Single-use packaging was dominant in lunchboxes due to the presence of snack food items, and vegetables were the least preferred food group, as indicated by higher food waste. Given that school-based dietary trends of children are consistent with previous research, reducing waste in school lunchboxes can potentially dovetail with public health nutrition goals. There is utility in studying the current school food environment to guide the development of school-based programs and interventions, in particular interventions that improve the quality of foods brought from home to school, not just for children’s health but also for the environment. Understanding the multiple determinants of parental (or even children’s) lunchbox packing behaviour is critical to understand the barriers and facilitators to packing an environmentally friendly lunchbox for improved health and environmental outcomes. Future research can also examine the extent to which children are responsible for packing their own lunches. There is also potential to further mobilise intersectionality of health and sustainability in school food policies and programmes.

## Supporting information

Lalchandani et al. supplementary material Lalchandani et al. supplementary material

## Supplementary material

For supplementary material accompanying this paper visit https://doi.org/10.1017/S1368980025000126
