# Supplementary material for: Sneak peek: food, waste and packaging characteristics of South Australian school children’s lunchboxes
Source: Public Health Nutr. 2025 Feb 3;28(1):e52. doi: 10.1017/S1368980025000126 (PMC11984001; doi:10.1017/S1368980025000126)
Supplement: Lalchandani et al. supplementary material [file S1368980025000126sup001.docx]

**Supplementary I: Audit details for each site, including school type, socio-economic status (SES), and fruit fly outbreak area in South Australia**

| **Audit number** | **Audit date** | **Audit day** | **School type** | **School SES** | **Fruit fly outbreak area** |
| --- | --- | --- | --- | --- | --- |
| 1 | 15-Mar-21 | Monday | Preschool | High | Yellow |
| 2 | 01-Apr-21 | Thursday | Primary School | Low | Green |
| 3 | 08-Jun-21 | Tuesday | Preschool | High | Green |
| 4a | 11-Jun-21 | Friday | Preschool | Med | Yellow |
| 4b | 28-Jun-21 | Monday |  |  |  |
| 5 | 16-Jun-21 | Wednesday | Primary School | High | Yellow -> Red |
| 6 | 17-Jun-21 | Thursday | Preschool | High | Green |
| 7 | 21-Jun-21 | Monday | Preschool | High | Yellow |
| 8 | 23-Jun-21 | Wednesday | Preschool | Med | Yellow -> Red |
| 9 | 23-Jun-21 | Wednesday | Primary School | Med | Yellow -> Red |
| 10 | 24-Jun-21 | Thursday | Preschool | Med | Yellow |
| 11 | 29-Jun-21 | Tuesday | Preschool | Med | Green |
| 12 | 30-Jun-21 | Wednesday | Primary School | Low | Red |
| 13 | 04-Aug-21 | Wednesday | Preschool | Low | Green |
| 14 | 06-Aug-21 | Friday | Preschool | Low | Green |
| 15 | 06-Aug-21 | Friday | Primary School | Low | Green |
| 16 | 09-Aug-21 | Monday | Preschool | High | Yellow |
| 17 | 10-Aug-21 | Tuesday | Preschool | Low | Green |
| 18 | 17-Aug-21 | Tuesday | Preschool | Low | Yellow |
| 19 | 19-Aug-21 | Thursday | Primary School | High | Yellow |
| 20 | 20-Aug-21 | Friday | Primary School | Low | Green |
| 21 | 02-Sep-21 | Thursday | Preschool | Med | Yellow |
| 22 | 09-Sep-21 | Thursday | Primary School | Med | Yellow |
| 23 | 13-Sep-21 | Monday | Primary School | High | Red |

Note: Red (Restrictions apply); Yellow (Restrictions apply); Green (No outbreak restrictions apply while remaining inside this area) | Based on fruit fly outbreak map from <https://fruitfly.sa.gov.au/outbreak-map>
